# E-*β*-Ocimene, a Volatile Brood Pheromone Involved in Social Regulation in the Honey Bee Colony (*Apis mellifera*)

**DOI:** 10.1371/journal.pone.0013531

**Published:** 2010-10-21

**Authors:** Alban Maisonnasse, Jean-Christophe Lenoir, Dominique Beslay, Didier Crauser, Yves Le Conte

**Affiliations:** INRA, UMR 406, Abeilles et Environnement, Laboratoire Biologie et Protection de l'Abeille, Avignon, France; Centre de Recherches su la Cognition Animale - Centre National de la Recherche Scientifique and Université Paul Sabatier, France

## Abstract

**Background:**

In honey bee colony, the brood is able to manipulate and chemically control the workers in order to sustain their own development. A brood ester pheromone produced primarily by old larvae (4 and 5 days old larvae) was first identified as acting as a contact pheromone with specific effects on nurses in the colony. More recently a new volatile brood pheromone has been identified: E-*β*-ocimene, which partially inhibits ovary development in workers.

**Methodology and Principal Finding:**

Our analysis of E-*β*-ocimene production revealed that young brood (newly hatched to 3 days old) produce the highest quantity of E-*β*-ocimene relative to their body weight. By testing the potential action of this molecule as a non-specific larval signal, due to its high volatility in the colony, we demonstrated that in the presence of E-*β*-ocimene nest workers start to forage earlier in life, as seen in the presence of real brood.

**Conclusions/Significance:**

In this way, young larvae are able to assign precedence to the task of foraging by workers in order to increase food stores for their own development. Thus, in the complexity of honey bee chemical communication, E-*β*-ocimene, a pheromone of young larvae, provides the brood with the means to express their nutritional needs to the workers.

## Introduction

In eusocial insects, the brood is completely dependent on the care provided by the nurses. A lack of workers and especially nurse care lead to brood decline and death [1]. Thus, larval needs for food or warmth, as well as their age, need to be recognized by nurses, while the workers have to allocate energy to fulfil these needs.

In the honey bee *Apis mellifera*, there is evidence for a complex system of chemicals produced by the larvae to adjust the behaviour and the physiology of workers to the needs of the brood [2], [3], [4], [5]. Brood pheromones with primer and releaser effects on workers have been identified [5], [6]. One pheromone named brood ester pheromone (BEP), is composed of a blend of 10 methyl and ethyl esters [7]. This pheromone, modulates the feeding and pollen foraging behaviour of workers [8], [9], [10], inhibits the activation of the worker ovary [11], [12], [13], induces workers to cap brood cells [7] and increases the rate of protein production in the hypopharyngeal glands of workers [14], [15]. In addition, BEP modulates the behavioural maturation of honey bee workers [16], then inducing workers to take care of the brood rather than allocating energy to outside activities. Due to the relatively low volatility of the esters, this larval signal can be assimilated as a contact pheromone that targets nurse workers directly involved in feeding the larvae.

Recently, we identified another brood pheromone component, E-*β*-ocimene, acting as a primer pheromone by inhibiting maturation of workers ovaries [17]. This highly volatile molecule emitted by the brood is easily dispersed within the colony. Subsequently all workers on the nest can be in direct contact with this signal, such as the nurse (young bee), but also the middle-aged bees, from ages 12–21 days, that specialize on nectar processing and nest maintenance but show no interest in brood care [18]. Thus we hypothesize that E-*β*-ocimene could act as a signal targeting the different worker castes and could induce changes in colony-wide social regulation complementarily to BEP.

Here, we studied the emission of E-*β*-ocimene and its effects on worker physiology and behaviour to test if this brood pheromone acts as a signal in the social regulation of workers.

The E-*β*-ocimene emission was measured for all immature instars (larvae to pupae) to supplement previous data from Maisonnasse et al. (2009) [17]. Before the analyses each individual was weighed in order to calculate the E-*β*-ocimene emission per mg of weight. Then we compared immature E-*β*-ocimene production to previously published BEP immature emission [19] in order to determine if the two pheromone profiles overlap during immature development.

We also studied the action of E-*β*-ocimene on nurses. Nurses secrete 60 to 80% of the brood diet from their developed hypopharyngeal glands, providing a secretion rich in protein for young larvae [20]. The larvae can stimulate the development of these glands in nurses in order to consume a diet richer in protein [21]. We investigated whether E-*β*-ocimene could mediate an increase in the size and protein production of the hypopharyngeal glands of nurses, like that seen with BEP, for produce a food richer in protein that would assure better development of larvae.

Finally, we analysed the global action of E-*β*-ocimene in social regulation. In particular, the brood can modify the division of labour of workers, by accelerating the behavioral development of workers, inducing worker nest bees to become foragers earlier in life [16], [22], [23]. In addition, the larvae manipulate the ratio of worker nest bees to foragers to increase their individual fitness. The volatility of E-*β*-ocimene allows for a distribution of pheromone to the nurse bees as well as to middle-aged bees. Thus we tested if larvae, by emitting E-*β*-ocimene, could modify behavioural maturation of workers.

## Materials and Methods

### E-*β*-ocimene quantification in immature honey bees

E-*β*-ocimene emitted by different larval and pupal instars was sampled by solid phase microextraction. A 65 µm Carbowax was used as in a previous study [17] to identify E-*β*-ocimene. No peak co-elution occurs with this fiber. The analyses were done on three different hives (*Apis mellifera* sp.) from an apiary controlled with standard maintenance procedure. After removing a frame from the hive, larvae and pupae were cautiously picked and delicately enclosed in 15 mL glass vials. Each sample was weighed before analysis to assess the mean weight of larvae and pupae. Aerial emissions were analyzed for groups of 20 larvae at stage L1 (newly hatched larvae) and L2-3 (2 and 3 days old larvae) while individuals at stage: L4-5 (4 and 5 days old larvae), pre-pupae and pupae (white, pink and black eyes) were grouped by 10 due to their higher body volume. Fiber adsorption lasted 20 min in an incubator at 34°C and 50% humidity before immediate desorption into a gas chromatograph (Varian-Chrompack 4000, USA, CPSil 8 CB-MS 30 m×0.25 mm column) with the following parameters: column temperature 40°C for 2 min, 40°C to 200°C at 30°C.min^−1^, and a final step from 200°C to 320°C at 10°C.min^−1^. A clean empty vial was used as a control sample to be certain that E-*β*-ocimene originated from the bees. The amount of E-*β*-ocimene of each sample was determined by comparison to a standard curves obtained by analysing known quantities of external standard (E-*β*-ocimene, International Flavors & Fragrances, Spain) in the same conditions. A minimum of 4 groups of each stage were sampled. The confirmation of E-*β*-ocimene was done by a mass spectrometer. The GC–MS system was a Polaris ion-trap mass spectrometer/Trace 2000 GC (ThermoQuest). The mass spectrometer was operated in the electron impact mode at 70 eV with continuous scans (every 0.2 sec) from a mass to charge ratio (m/z) of 60 to 520.

### E-*β*-ocimene effect on workers hypopharyngeal glands development

The same methods were used for the E-*β*-ocimene treatment and experimental design in Maisonnasse et al. (2009) [17]. Briefly, groups of 100 emergent bees from a mix of 4 hives were introduced randomly into 10 cages for treatment and 10 cages for control (Pain cages: 11×8.5×5.8 cm, [24]) and separated into two incubators (33°C, 60% RH) to prevent E-*β*-ocimene dispersion. Bees were kept in the dark and fed with a honey-sucrose candy, fresh pollen (to promote worker glands development [25]), and water *ad libitum*, from emergence until 14 days of age. For the treatment, the compound was mixed with paraffin oil to allow it to slowly dissipate over 24 hours. The instars L2-3 emitted the higher rate of E-*β*-ocimene, therefore we assumed that these instars had a more significant effect in the nest. In addition the nurse bees frequently visit cells [3]. Thus we employed a 10 larvae (L2-3) equivalent/caged bee (10 µg of E-*β*-ocimene/bee, 1000 µg of E-*β*-ocimene/cage) and only paraffin oil for the control. The E-*β*-ocimene and paraffin mix or paraffin only were placed in a wire screen-covered petri-dish at the bottom of the cage so that the bees could never be in direct contact with the petri-dish. The treatment was performed daily in the morning by the replacement of the petri-dish by a new one with the correct treatment. We confirmed the evaporation of 1000 µg of E-*β*-ocimene per cage in 24 hours by sampling, at 3, 6, 12 and 24 hours, the remaining amount of E-*β*-ocimene in a Pain cage at 34°C (same conditions as the experiment). The quantity of E-*β*-ocimene release by the solution mixture is high in the cage during the 6 first hours and decreases after 12 hours; at 24 hours only a small quantity of E-*β*-ocimene is found in the cage.

For each cage, the hypopharyngeal glands were removed from 10 randomly selected bees on day 14 which is the day when the workers have the highest level of protein in these glands [15]. The size of the acini changes with hypopharyngeal glands development, growing until day 6, stabilizing during days 6 to 14, when workers are known to feed the larvae with royal jelly [26], and decreasing from day 15, until the size of the glands in foragers resembles that of the undeveloped gland of newly emerged bees. The workers glands were removed with forceps though an incision in the front part of the head. The level of gland activation was analysed by dissecting microscope as the size of the gland is positively correlated with gland activity [27]. The level of gland development was determined using a 5 point scale classification (undeveloped gland: 1 to full developed gland: 5; usually nurses have a level of gland activation around 2.5 – 3.5 and foragers 1 – 1.5 on average). The total hypopharyngeal glands protein content was also assessed using the Bradford method [28] for each bee because a higher protein level in the glands gives higher quality nourishment for the larvae. Protein levels were measured via spectrophotometer at 595 nm and compared to the standard albumin. The hypopharyngeal glands score and the Bradford assay were done by a blind test to ensure impartiality in the results.

### E-*β*-ocimene effect on workers behavioural maturation

Honey bee experiments were performed in Avignon during June and July of 2008 and 2009. Standardized colonies, called “triple cohort colonies” were conducted to test the effect of E-*β*-ocimene on honey bee behavioural maturation [29]. Each colony was composed of 3 cohorts of bees: 500 nurses, 500 foragers and 500 one-day-old bees (*Apis mellifera* sp. honey bees from our apiary). The one-day-old bees were collected from honey bee combs containing the last nymphal stage, which were removed from the hive and placed in an incubator (33°C, 60% RH). One day after, emergent bees (one-day-old bees) appeared on the comb and were painted on the thorax (focal cohort). The foragers, bees with pollen loads (pollen foragers) or distended abdomen (nectar foragers), were captured when they returned to the hive by closing the hive entrance. The nurses, considered to be bees with their head inside a larval cell, were sampled from an open brood frame. For each trial the honey bees came from the same hives to lessen genetic variation. Each test colony was placed in a nucleus (small hive) with two half frames, one full of honey and one empty. In all nuclei, one plastic strip (Bee Boost, PheroTech, Canada) was installed containing the queen mandibular pheromone which mimics a queen (one queen pheromone equivalent released by day, [30]). This plastic strip minimizes the natural genetic and pheromonal variation of queens for laying eggs and workers for raising larvae. Workers in this small colonies show normal ontogeny of behaviour [29]. With these colonies, the age at which focal bees begin foraging is precisely know while keeping constant other potentially important variables, such as colony demography, genetic structure, or pheromone dispersion [31]. The age at first foraging of the focal cohort was determined by the age at which the first 50 bees of the focal cohort initiate foraging.

Each trial was formed by three test colony, the first received 10,000 larvae (L2-3) equivalent/day (high dose, equivalent to 20 larvae L2-3/focal bee or 20 µg of E-*β*-ocimene/focal bee), the second 5,000 larvae (L2-3) equivalent/day (low dose, equivalent to 10 larvae L2-3/focal bee or 10 µg of E-*β*-ocimene/focal bee) and the last one only paraffin oil (control). During the spring the queen can lay approximately 1500–2000 eggs per day [20], thus the number of larvae of stage L2-3 is around 3000 to 6000 individuals in the colony. The doses used in this experiment, 5,000 and 10,000 L2-3 larvae equivalent per day, are on a biological scale. Bees were exposed to chronic E-*β*-ocimene treatment and received E-*β*-ocimene through evaporation. Four trials were made in 2008 and four trials in 2009. All colonies were placed in a field isolated from others hives to avoid foraging competition and nuclei disturbance. The treatment started when one-day-old bees (focal cohort) were introduced in the nuclei. The E-*β*-ocimene was mixed in the paraffin. The mixture or paraffin oil alone were introduced into the bottom of the nuclei, in a petri-dish placed below a wire screen-covered; thus the bees could never be in direct contact with the molecule. The treatment was performed daily at 9h00 in the morning without disturbing the colony by removing the petri-dish through a special door behind the nucleus and replacing with another one with the appropriate treatment. Twice a day (once in the morning and once in the afternoon) each nucleus was closed for 45 to 60 min and the nectar or pollen foragers of the focal cohort, accumulated on the screen, were counted and additionally marked.

After 10 days of experimentation and at the end of the trial experiment, we made a census of the number of focal bees and of the total number of bees to prevent potential population variation (mortality) between each nucleus of each trial. Pictures of nuclei frames were taken and all the marked and un-marked bees from each nucleus were counted.

### Statistics

Mann–Whitney U tests were used to test differences in E-*β*-ocimene emission between the different immature instars and for E-*β*-ocimene effects on worker bee hypopharyngeal glands development. The results of E-*β*-ocimene influence on honey bee behavioural development were analysed with a two-ways ANOVA (years and treatments) followed by Fisher post-hoc test (STATVIEW 5.0, SAS Institute, Cary, NC).

## Results

### E-*β*-ocimene quantification

The production of E-*β*-ocimene per larva increased from larva stage L1 (3.9 ng/larva/20 min) to L2-3 (14.01 ng/larva/20 min) and decreased at stage L4-5 (0.42 ng/larva/20 min). These 3 groups produced significantly different amounts of E-*β*-ocimene (L1 vs L2-3, Z = −3.441, P<0.001; L1 vs L4-5, Z = −3.272, P<0.01 and L2-3 vs L4-5, Z = −3.435, P<0.001).

**Figure 1:**
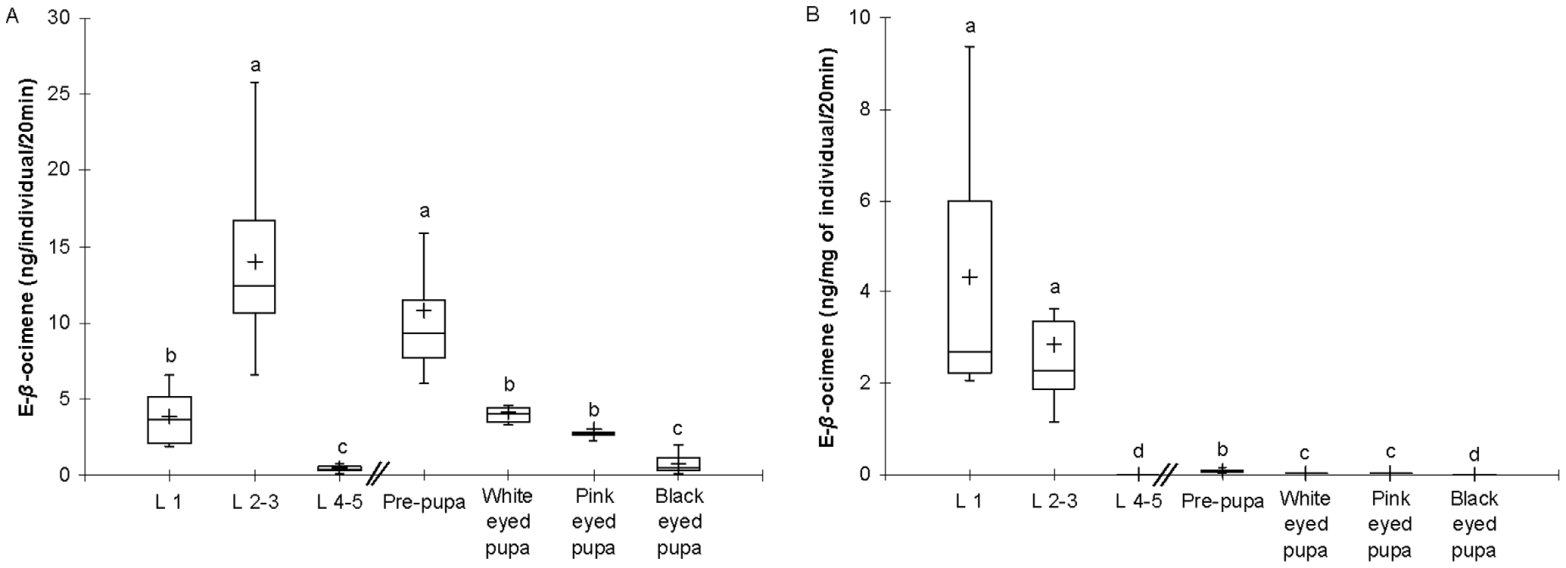
E-*β*-ocimene emission by the brood during 20 minutes. A. as ng per individual. B. as ng per mg of individual. (Different letters indicate significant differences in individual E-*β*-ocimene emission (P<0.05) (n≥4), cross: mean, box: 25%–75%; line in the box: median, whisker: Min-Max, //: larvae cell capping).

The amount of E-*β*-ocimene was high for pre-pupae stage (10.83 ng/individual/20 min), with no difference production of L2-3 stage (Z = −1.688, P = 0.0914). Production decreased in the white and pink eyed pupal stages (4.18 and 3.99 ng/pupa/20 min respectively) with no difference in production with stage L1 (Z = −0.293, P = 0.817 and Z = −2.84, P = 0.7697 respectively). The production declined in the black eyed pupal stage (0.77 ng/pupa/20 min) to a level similar to the production in stage L4-5 (Z = −0.571, P = 0.5657) (Fig. 1A).

The production of E-*β*-ocimene per mg of individual gave different results. According to their weight the smaller individuals (L1-2-3) produced the highest quantity of E-*β*-ocimene: L1 = 4.33 ng/mg of larva/20 min and L2-3 = 2.84 ng/mg of larva/20 min with no significant difference between them (Z = −1.033, P = 0.3017). The other immature groups produced significantly less E-*β*-ocimene (P<0.01) with a maximum of 0.085 ng/mg per individual/20 min (Fig. 1B).

### E-*β*-ocimene and workers hypopharyngeal glands development

The level of hypopharyngeal glands development was not significantly different between the honey bees treated with the E-*β*-ocimene (2.52±0.07) and the control bees (2.72±0.07) (Z = −0.507, P = 0.6120). The mean protein amount produced by the hypopharyngeal glands was not different between the control group (57.21±3.16 µg/bee glands) and the E-*β*-ocimene treated group (51.04±2.35 µg/bee glands) (Z = −1.293, P = 0.1961).

### E-*β*-ocimene and workers behavioural maturation

**Figure 2:**
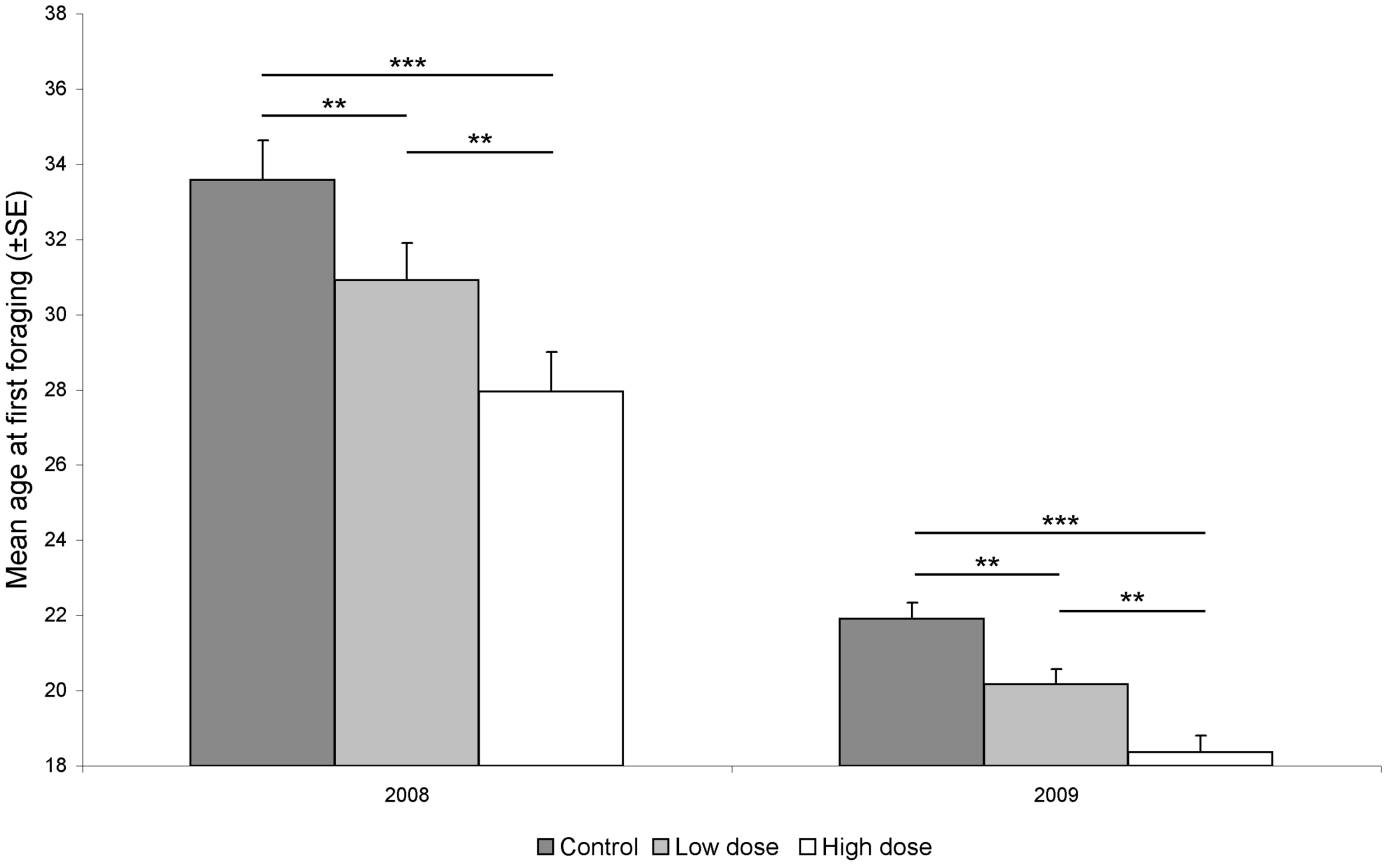
Effect of high and low E-*β*-ocimene doses on worker age at first foraging. (10 000 larvae and 5000 larvae (stage 2-3) equivalent/day/nuclei respectively, mean age at onset of foraging of the focal cohort for each treatment ± Standard Error, * denotes significant differences: *** P<0.001, ** P<0.01).

No significant variation in the total population (census) was found among focal cohorts within any trial. The ANOVA test revealed significant treatment and years effect and no interaction (Year: F_1,1119_ = 318.5 P<0.001, Treatment F_2,1119_ = 19.6 P<0.001, Interaction F_2,2399_ = 1.0 P = 0.3615). Exposure to E-*β*-ocimene accelerated the mean age at onset of foraging for both low and high dose groups (P<0.01 and P<0.001 respectively) compared to the control group. Bees with low and high doses of E-*β*-ocimene started to forage earlier in 7 of 8 trials. The high dose of E-*β*-ocimene exerted a stronger effect, causing a significantly younger age at the onset of foraging relative to the low dose (P<0.01) (Fig. 2).

## Discussion

In presence of brood, workers initiate foraging earlier compared to broodless workers [16], [22], [23]. Our results clearly show that E-*β*-ocimene is a component of the signal emitted by the brood accelerating the age at the onset of foraging for workers with no effect on hypopharyngeal glands activity. Thus, E-*β*-ocimene induces workers earlier into the task of foraging, thereby optimizing food collection and processing for the colony as well as for larval feeding. E-*β*-ocimene is a primer pheromone with two actions on workers physiology: inhibition of worker ovaries [17] and acceleration of workers behavioural maturation (this paper).

At social level, E-*β*-ocimene is a compound controlling honey bee behavioural maturation within a complex process that ultimately maintains colony homeostasis. An overabundance of foragers leads to a lack of nurses in the colony, and thus a decline in brood care; conversely too many nurses cause a decrease in food storage in the colony and a subsequent decline in food for brood nourishment due to the scarcity of foragers. Consequently, a proper nurse-forager ratio is key to maintaining honey bee social homeostasis. Therefore, the regulation of honey bee behavioural maturation must be highly controlled, most likely through a colony-level feedback network. Queen, old brood and foragers produce pheromones, the Queen Mandibular Pheromone [32], BEP at high doses [16] and ethyl oleate [33] respectively, which slow down the progression of young bees towards the tasks typical of older bees. But young larvae also have something to say. In producing E-*β*-ocimene and low doses of BEP [16], they have the opposite effect of old brood on bee maturation, which is to accelerate worker age at first foraging. In this way, worker maturation occurs in a complex milieu of pheromonal compounds. Considering that workers adjust their behavioural development in response to specific pheromones in the nest [16], modification of pheromone production by the queen or the brood could change the pheromone level in the hive and trigger variation in worker maturation. Alternatively, the different pheromones could target different worker castes. For example, the middle-aged bees' transition to foragers is important for maintaining the dynamic caste ratio [18]. E-*β*-ocimene could be the signal for the transition of middle-aged bees to the forager caste, while BEP could slow the transition from nurse to the middle-aged bee caste. Further experiments are needed to understand the direct effect of larvae, but also queen and worker signals, on the middle-aged bee group. These complementary experiments integrating other important factors, like pheromone dose or role of a specific caste, should be done to resolve the question of the complex role of pheromones in honey bee social regulation.

**Figure 3:**
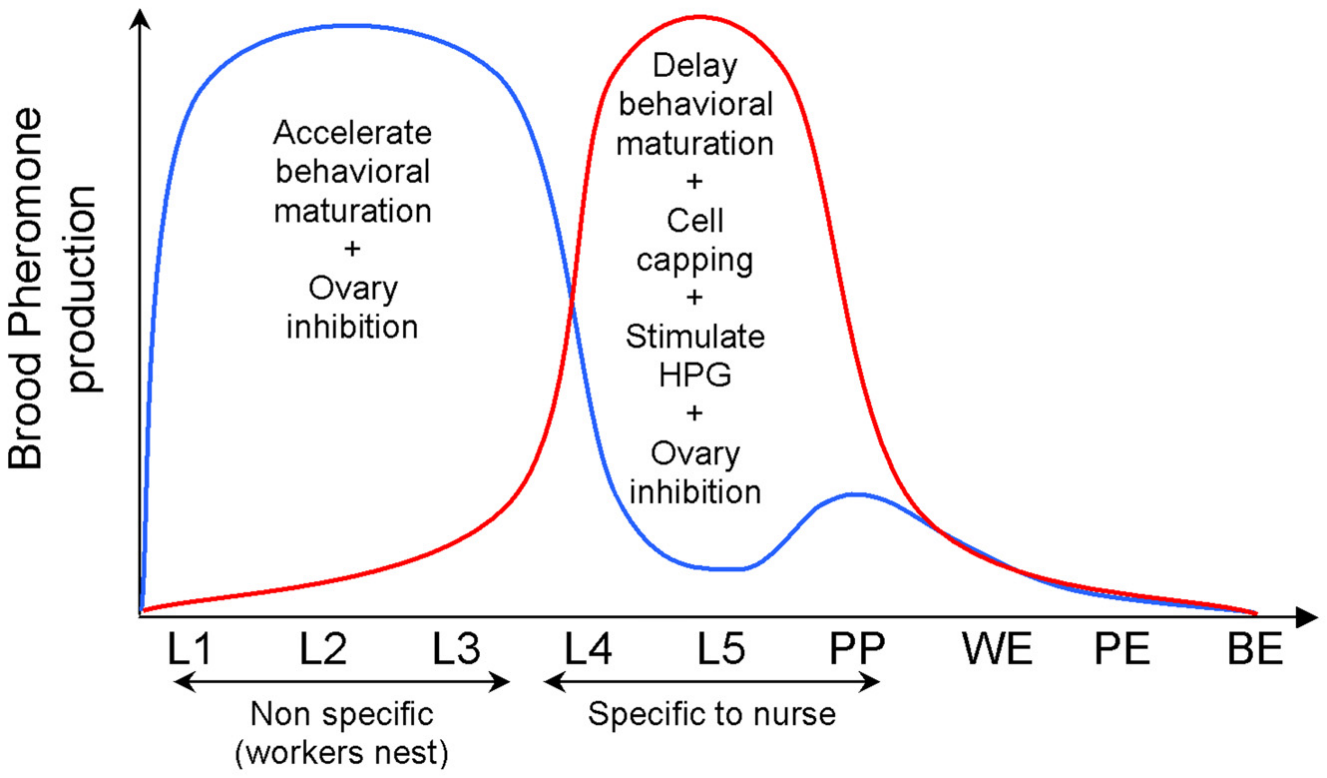
Production of BEP (red) and E-*β*-ocimene (blue) per mg of the different brood instars and their effects on worker physiology and behaviour. (L1 to L5, pre-pupae PP, pupae white eyes WE, pink eyes PE, black eyes BE).

At individual level the young and the old larvae emit different quantities of pheromones that have different volatilities. Taken as the amount of compound produced per gram of larvae, E-*β*-ocimene is emitted principally by the young instars (L1, L2-3) while BEP reaches a maximum value during the capping stage (L4-5) [19] (Fig. 3). E-*β*-ocimene (boiling point 73°C), which belongs to the terpene family, is volatile so it has an aerial transmission (targeting all worker castes), while the BEP (boiling point around 200°C), which belongs to the ester family, has a low volatility which is transmitted by contact (target workers close to the larvae cells).

**Figure 4:**
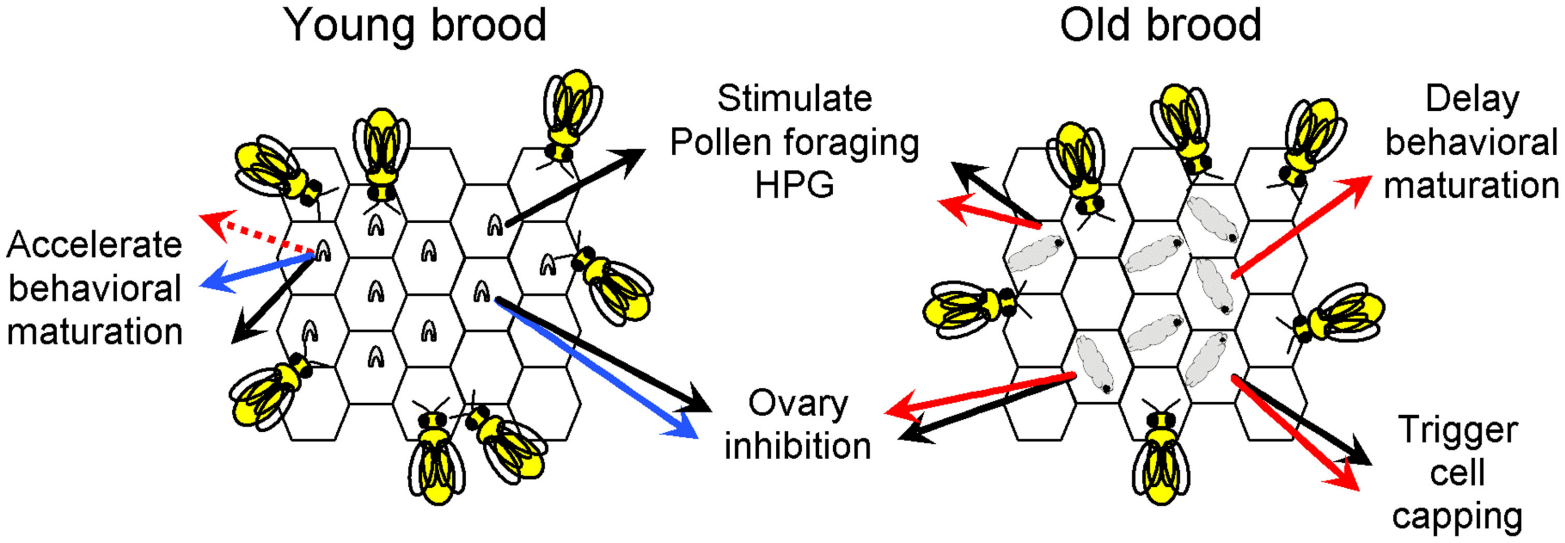
Comparison of the different effects of BEP (red), E-*β*-ocimene (blue) and real brood (black) on worker physiology and behaviour. (dotted line: low pheromone production).

These two specific pheromonal signals have opposing effects on workers (Fig. 3,4). By emitting a low quantity of BEP and a great amount of E-*β*-ocimene, young larvae are able to accelerate the age at onset of foraging of worker bees [16], [34]. In contrast, old larvae inhibit honey bee behavioural development by producing a high quantity of BEP [16]. Thus young and old larvae play opposite roles in the behavioural maturation of worker bees according to their specific needs. The young larvae promote foraging (low need in nurses) and old larvae promote tending (high need in nurses). We then presume that young larvae need less attention from nurses than old larvae. For instance, young and old larvae do not have the same workers needs. When worker eggs hatch, young larvae are provided with royal jelly from the mandibular and hypopharyngeal glands of the nurses until they reach an age of 3.5 days old [20]. Afterwards, old larvae receive brood food, a mixture principally made of the nurse's hypopharyngeal glands secretions, honey and pollen. This brood food mixture is given to old larvae by the nurses in higher quantities [20], [35], [36]. After food requirement, old larvae need nurses to help in capping their cells, and then, still require nurses and hive workers for their thermoregulation, as they are very sensitive to cooling [37].

As a consequence, old larvae by emitting BEP, keep nurses in contact with them for a longer time, develop worker hypopharyngeal glands [14], [15], [34] and engage them in specific tasks like capping cells, nourishment or tending [7], [8], [38], [39]. On the contrary, young larvae by producing E-*β*-ocimene, accelerate worker maturation (workers become foragers earlier in life) thereby optimizing foraging and food collection.

Thus we can consider BEP as a “specific worker caste signal”, with a specific and local action in the colony: the tending of old larvae. And then, we propose E-*β*-ocimene as a “non-specific worker castes signal” with a global action on the colony: increasing food provision.

Therefore, by emitting E-*β*-ocimene and BEP, the young and old larvae signals are involved in enforcing different worker tasks (Fig. 4); nevertheless they also have a common action in the nest: the inhibition of worker ovary activation [12], [17]. This plays a major role in the productivity of the nest because as reproductive workers do not work as hard as sterile workers [40], showing a reduction in both tending to larvae and foraging tasks, which decreases the inclusive fitness of the colony. E-*β*-ocimene and BEP both partially inhibit the worker ovary activation, and a possible synergistic interaction needs to be tested. In addition, workers can escape from the reproductive control induces by queen and brood [41]. But the difference between E-*β*-ocimene and BEP in their temporal production, mode of transmission and targets could be a strong barrier against the development of reproductive workers in the colony by decreasing their potentiality to bypass pheromonal control. According to theories of social insect communication, an honest signal is expected to be relatively simple while a complex signal would indicate the presence of a coercive force between sender and receiver [42], [43]. In this way by using two pheromones larvae would repress the activation of the workers ovaries. By definition coercion in social *Apis mellifera* is a form of pressure to prevent workers from acting selfishly and thereby harming group or colony as a whole [44]. In *Apis mellifera* workers are frequently coerced into acting altruistically [44]: workers repress reproduction of other workers through egg eating and aggression (policing) [45], [46]. Thus the coercion of laying workers bees to remain sterile comes from policing by other workers, but also from pheromones of the larvae and queen. This colony-level coercion, or colony arms race, against reproductive workers would benefit the group and increase its inclusive fitness. Therefore despite using a dishonest signal in the nest to control reproduction, this communication seems to serve the entire society.

The production of two different types of pheromones by the larvae, gives a powerful signal to adjust all workers for colony tasks, especially larval care. E-*β*-ocimene is a young larval pheromone, highly volatile, and easily dispersed within the colony for a large scale action while BEP is an old larval pheromone with low volatility, spread by contact, with a precise action. The complementary effect of these pheromonal components supports also the hypothesis that a special chemical syntax exists in the colony for fine-tuning social regulation. It also confirms the remarkable and unexpected complexity of honey bee pheromonal communication.
